# AMP-Activated Protein Kinase (AMPK) Mediates Nutrient Regulation of Thioredoxin-Interacting Protein (TXNIP) in Pancreatic Beta-Cells

**DOI:** 10.1371/journal.pone.0028804

**Published:** 2011-12-14

**Authors:** Maayan Shaked, Mali Ketzinel-Gilad, Erol Cerasi, Nurit Kaiser, Gil Leibowitz

**Affiliations:** Endocrinology and Metabolism Service, Department of Medicine, Hadassah - Hebrew University Medical Center, Jerusalem, Israel; University of Bremen, Germany

## Abstract

Thioredoxin-interacting protein (TXNIP) regulates critical biological processes including inflammation, stress and apoptosis. TXNIP is upregulated by glucose and is a critical mediator of hyperglycemia-induced beta-cell apoptosis in diabetes. In contrast, the saturated long-chain fatty acid palmitate, although toxic to the beta-cell, inhibits TXNIP expression. The mechanisms involved in the opposing effects of glucose and fatty acids on TXNIP expression are unknown. We found that both palmitate and oleate inhibited TXNIP in a rat beta-cell line and islets. Palmitate inhibition of TXNIP was independent of fatty acid beta-oxidation or esterification. AMP-activated protein kinase (AMPK) has an important role in cellular energy sensing and control of metabolic homeostasis; therefore we investigated its involvement in nutrient regulation of TXNIP. As expected, glucose inhibited whereas palmitate stimulated AMPK. Pharmacologic activators of AMPK mimicked fatty acids by inhibiting TXNIP. AMPK knockdown increased TXNIP expression in presence of high glucose with and without palmitate, indicating that nutrient (glucose and fatty acids) effects on TXNIP are mediated in part *via* modulation of AMPK activity. TXNIP is transcriptionally regulated by carbohydrate response element-binding protein (ChREBP). Palmitate inhibited glucose-stimulated ChREBP nuclear entry and recruitment to the Txnip promoter, thereby inhibiting Txnip transcription. We conclude that AMPK is an important regulator of Txnip transcription *via* modulation of ChREBP activity. The divergent effects of glucose and fatty acids on TXNIP expression result in part from their opposing effects on AMPK activity. In light of the important role of TXNIP in beta-cell apoptosis, its inhibition by fatty acids can be regarded as an adaptive/protective response to glucolipotoxicity. The finding that AMPK mediates nutrient regulation of TXNIP may have important implications for the pathophysiology and treatment of diabetes.

## Introduction

Beta-cell dysfunction is the hallmark of type 2 diabetes [1]. Little is known about the mechanisms that initiate type 2 diabetes; however, hyperglycemia and elevated free fatty acids (FFA) play an important role in the progression of beta-cell dysfunction in diabetes, a process called glucolipotoxicity [2]. The mechanism of beta-cell glucolipotoxicity, while not entirely clear, is believed to involve oxidative and ER stress [3], [4]. Hyperglycemia and FFA act in concert to amplify the stress response in beta-cells, resulting in beta-cell dysfunction and apoptosis. However, glucose and FFA differ in their regulation of the various stress pathways. As an example, glucose-induced beta-cell apoptosis results mainly from oxidative stress with stimulation of the intrinsic mitochondrial death pathway, whereas ER stress plays a central role in FFA-induced beta-cell apoptosis [3], [5]. The divergent effects of glucose and FFA on oxidative stress could result from differential regulation of the thioredoxin system [6].

Thioredoxin (TRX) is a ubiquitous oxidoreductase, which is highly expressed in pancreatic β-cells [7]. TRX partners with thioredoxin reductase and thioredoxin peroxidase to reduce oxidized proteins and scavenge free radicals [8]. Thioredoxin-interacting protein (TXNIP), a member of the arrestin family, binds to the redox-active cysteine residues of TRX and inhibits its oxidoreductase activity, thus functioning as an endogenous inhibitor of TRX [9]. Of relevance to the fate of beta-cells in diabetes, TXNIP expression is robustly induced by glucose in islets [10], [11], [12]. Glucose regulates TXNIP by increasing the binding of the carbohydrate response element-binding protein (ChREBP) to the Txnip promoter, with recruitment of P300 and histone H4 acetylation, thereby stimulating Txnip transcription [11]. Islets from TXNIP mutant mice, and beta-cells in which TXNIP was knocked-down, are protected from glucose-induced apoptosis [6], [10], [12]. Moreover, TXNIP deficiency was shown to prevent beta-cell apoptosis and hyperglycemia in rodent models of type 2 diabetes [13]. Thus, TXNIP is emerging as a critical link between hyperglycemia and beta-cell death in diabetes.

Contrasting the glucose stimulation of TXNIP, the saturated long-chain fatty acid palmitate, which is a strong inducer of beta-cell apoptosis, inhibits TXNIP expression [6]; however, the mechanisms involved are largely unknown. AMPK is an important nutrient sensor regulating FFA metabolism in various tissues including pancreatic beta-cells [14], [15]. Furthermore, it was shown to modulate the activity of various proteins and transcription factors through phosphorylation [16]. In light of the central role of ChREBP in the regulation of TXNIP and of AMPK in lipid sensing and metabolism [14], we investigated whether AMPK and ChREBP are involved in FFA inhibition of TXNIP in beta-cells.

## Methods

### Islet isolation and INS-1E beta-cell line culture

Islets were isolated from Wistar rats by collagenase digestion (Collagenase P; Roche Diagnostics GmbH, Mannheim, Germany), as described [17]. The islets were used after repeated washes with Hanks' balanced salt solution, and incubated overnight in RPMI 1640 medium (Biological Industries, Beit-Haemek, Israel) containing 5.5 mmol/l glucose with 10% fetal bovine serum, 100 U/ml penicillin, 100 mcg/ml streptomycin, and 2 mmol/l L-glutamine (Biological Industries). They were then taken for further incubations as described in *Experimental protocols*. Animal use was approved by the Institutional Animal Care and Use Committee of the Hebrew University and the Hadassah Medical Organization (approval number: MD-08-11428-4).

The rat insulinoma cell line INS-1E (kindly provided by Dr. Walker, The Weizmann Institute of Science, Rehovot, Israel) was used to study the mechanisms of nutrient regulation of TXNIP in beta-cells. INS-1E beta-cells were grown in RPMI 1640 medium containing different glucose concentrations as indicated, and supplemented with 10% fetal bovine serum, 100 U/ml penicillin, 100 mcg/ml streptomycin, 1 mmol/l sodium pyruvate, 2 mmol/l L-glutamine, 10 mmol/l HEPES and 0.05 mmol/l 2-mercaptoethanol.

### Experimental protocols

INS-1E cells and rat islets were incubated in RPMI medium with 0.55% (w/v) BSA with or without palmitate at various glucose concentrations for different periods of time, as indicated in the Figure legends. The palmitate-BSA solution was prepared as described [18]. Briefly, the sodium salt of palmitic acid was dissolved at a concentration of 10 mmol/l in 11% fatty acid- and endotoxin-free BSA in a shaking water bath at 37°C for 16 h. The pH was adjusted to 7.4 with 1 N NaOH, and the solution filtered through a 0.2 micrometer filter and stored at −20°C. Different dilutions of the palmitate-BSA solution were used as indicated in the Figure legends. In some experiments, INS-1E cells were treated with oleate, or non-metabolizable palmitic acid MEDICA analogs [19] (kindly provided by Dr. Rachel Hertz, the Hebrew University, Jerusalem, Israel). The effect of AMPK activation on TXNIP expression was studied by treating INS-1E cells and islets with metformin or aminoimidazole-carboxamide ribonucleotide (AICAR). All reagents were purchased from Sigma (Rehovot, Israel).

### AMPKalpha1 and AMPKalpha2 knockdown

Knockdown of AMPK isoforms was performed by transient transfection of 50 nmol/l small interfering RNA (siRNA) oligos for AMPKalpha1 and AMPKalpha2 (Dharmacon L-091373 and L-100623, Lafayette, CO) to INS-1E cells, plated in 12-well plates and grown overnight to approximately 50–70% confluence. Scrambled siRNA oligos (AllStars Negative Control, Qiagen, Basel, Switzerland) served as negative control. Transfection was performed in serum-free RPMI medium using JetPRIME (PolyPlus transfection, Illkirch, France) according to the manufacturer's instructions. Twenty-four hours after transfection the medium was replaced with the regular culture medium, and after additional 48 h the cells were incubated overnight at different glucose concentrations with and without palmitate or metformin, followed by extraction and analysis for the expression of AMPK isoforms and TXNIP.

### Quantitative real-time RT-PCR

RNA was extracted from INS-1E cells using Bio Tri RNA (Biolab, Jerusalem, Israel). Samples of 1 mcg total RNA were reverse-transcribed using Moloney murine leukemia virus reverse transcriptase (Promega, Madison, WI). Quantitative real time RT-PCR (qPCR) for TXNIP, AMPKalpha1 and AMPKalpha2 was performed with ABI PRISM 7900HT Sequence Detection System using the TaqMan Gene Expression Assay (Applied Biosystems, Foster City, CA). All samples were analyzed in triplicate; 18S ribosomal subunit (Applied Biosystems) was used as internal control.

### Transient and stable Txnip promoter transfection experiments

INS-1E cells were plated in 24-well plates and grown overnight to approximately 70% confluence. Cells were cotransfected with a luciferase reporter construct encoding the human Txnip promoter region 1777 bp upstream of the ATG start codon (FL) or with its carbohydrate response element (ChoRE) (D4) [11] (kindly provided by Dr. Anath Shalev, University of Wisconsin-Madison, Madison, WI), and with Renilla luciferase reporter plasmid (Biological Industries). Transfection was performed in serum-free RPMI using lipofectamine (Invitrogen). Six hours after transfection the medium was replaced and transcriptional activity assessed following an overnight incubation in medium containing different glucose concentrations without and with palmitate or metformin. In part of the experiments, the effects of different treatments on Txnip transcription were assessed using a stable reporter cell line: INS-1E beta-cells were stably transfected with constructs harboring luciferase under the control of the full-length Txnip promoter and pcDNA3 plasmid, which contains a neomycin resistance gene. Stable INS-1E beta-cell lines were generated by antibiotic selection following transfection; luciferase activity was determined using the luciferase assay kit (Promega).

### Chromatin immunoprecipitation (ChIP)

ChIP asssays were performed using a ChIP assay kit (SimpleChIP™, Cell Signaling, Boston, MA) according to the manufacturer's instructions. Lysates were prepared from 10^8^ cells per each treatment. Immunoprecipitation was performed overnight at 4°C with 4 mcg of anti-ChREBP antibody (Santa Cruz Biotechnology, Santa Cruz, CA) or with Normal Rabbit-IgG antibody, used as a negative control (SimpleChIP™, Cell Signaling). The purified DNA fragments were quantified by real-time PCR using the Power SYBR®Green system (Applied Biosystems) with primers corresponding to the Txnip ChoRE: sense, 5′-AAGGACCAAGTAGCCAATGGG; antisense, 5′- GTGCTGGCCCGGAGG.

### Immunofluorescence microscopy

INS-1E cells were plated on glass cover-slips pre-treated with poly-D-lysine (10 mcg/ml) (Sigma). The cells were treated for 4 h with 3.3 or 22.2 mmol/l glucose, with or without 0.5 mmol/l palmitate or 1 mmol/l metformin, and fixed in 4% paraformaldehyde (Biolab). Following permeabilization with 0.2% Triton X-100 (Sigma), the cells were incubated with goat antibody against human ChREBP (1∶50) (Santa Cruz Biotechnology); the secondary antibody for detection was rhodamine-conjugated donkey anti-goat antibody (1∶200) (Jackson ImmunoResearch Laboratories, West Grove, PA). Mounting medium containing DAPI (Vector Laboratories, Inc., Burlingame, CA) was used for nuclear counterstaining and the specimens were visualized with a Nikon E600 fluorescent microscope.

### Western blot analysis

Protein expression in whole cell and nuclear extracts was studied by Western blot using antibodies against TXNIP (MBL International Co, Woburn, MA), total AMPK, total and phosphorylated (Ser 79) acetyl CoA carboxylase (ACC) (Cell Signaling Technology, Beverley, MA), AMPKalpha1 and alpha2 (Upstate Biotechnology, Lake Placid, NY), ChREBP, Lamin B and GAPDH (Santa Cruz Biotechnology). Immunoreactive bands were visualized by chemiluminescence with ECL-Plus (Biological Industries). X-ray film densitometry was used for quantification (ImageMaster VDS-CL, Amersham Pharmacia Biotech, Buckinghamshire, UK). Immunoblots were scanned by ImageMaster and signals quantified using TINA Software.

### Apoptosis ELISA assay

INS-1E cells plated in 96-well plates were grown in RPMI 1640 containing 11.1 mmol/l glucose until reaching 70% confluence. The cells were then treated with 0.55% (w/v) BSA without or with increasing concentrations of palmitate at 11.1 and 22.2 mmol/l glucose for 16 h. They were then lysed and oligonucleosomes in the cytosol, indicative of apoptosis-induced DNA degradation, quantified using the Cell Death Detection ELISA^PLUS^ kit (Roche Diagnostics, Mannheim, Germany) according to the manufacturer's instructions.

### Data presentation and statistical analysis

Data shown are means ± SEM. Statistical significance of differences between groups was determined by one-way ANOVA followed by Newman-Keuls test using the InStat statistical program from GraphPad Software, Inc. (San Diego, CA). A paired-sample *t* test was used when the difference between a reference (taken as 100%) and test was analyzed. A *P* value of less than 0.05 was considered significant.

## Results

### Fatty acids inhibit TXNIP expression

**Figure 1:**
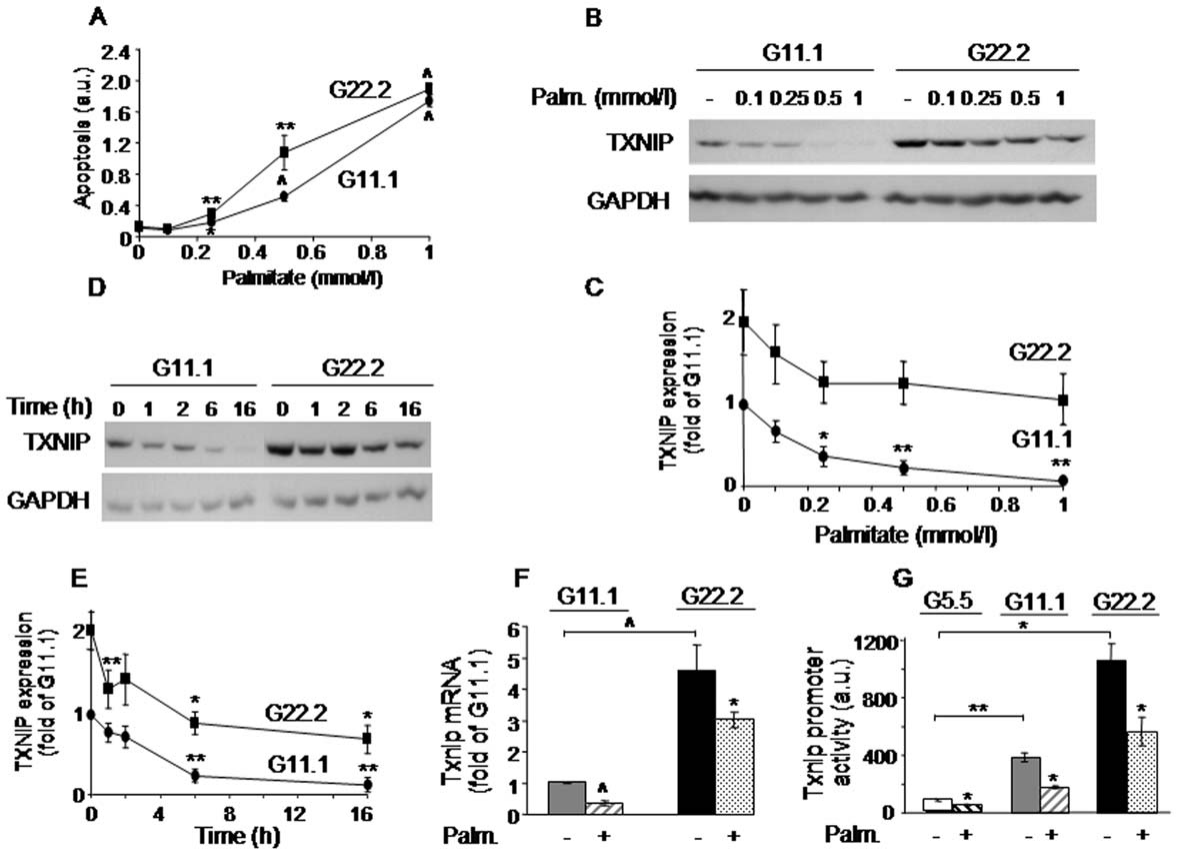
Palmitate effects on beta-cell apoptosis and TXNIP expression in INS-1E beta-cells. (A–C) INS-1E cells were incubated for 16 h with different concentrations of palmitate at 11.1 (G11.1) and 22.2 (G22.2) mmol/l glucose, as indicated. (A) Beta-cell apoptosis was analyzed using the Cell Death Detection ELISA^PLUS^ kit (Roche Diagnostics, Mannheim, Germany), as described in the Experimental procedures. (B, C) Concentration-dependent inhibition of TXNIP analyzed by Western blot. (D, E) Time-course of palmitate inhibition of TXNIP. INS-1E cells were incubated at 11.1 or 22.2 mmol/l glucose alone or with 0.5 mmol/l palmitate for the indicated time. TXNIP protein level was assessed by Western blot. Representative gels showing the expression of TXNIP (B, D), and quantification of TXNIP expression normalized to GAPDH (C, E) are presented. Results are expressed as fold of TXNIP expression at 11.1 mmol/l glucose (G11.1) in the absence of palmitate (n = 3). (F) Palmitate effect on Txnip mRNA. INS-1E cells were incubated in medium containing 11.1 or 22.2 mmol/l glucose with and without 0.5 mmol/l palmitate for 16 h. Txnip mRNA levels were analyzed by qPCR and corrected for the 18S rRNA, which served as an internal control. Results are normalized to Txnip mRNA levels at G22.2 without palmitate (n = 10). (G) Palmitate effect on Txnip gene transcription. Transcriptional activity was analyzed following transient transfection with a reporter construct encoding the human Txnip promoter region 1777 bp upstream of the ATG start codon, followed by 16 h incubation at 5.5, 11.1 and 22.2 mmol/l glucose. Results are normalized to transcriptional activity at G5.5 without palmitate. Data are expressed as means ± SEM (n = 4).* p<0.05, ** p<0.01, ∧ p<0.001 for the difference between the indicated groups (F, G) and untreated cells at the same glucose concentration (A, C, E), or between palmitate-treated and the paired untreated cells at the same glucose concentration (C, E, F, G).

**Figure 2:**
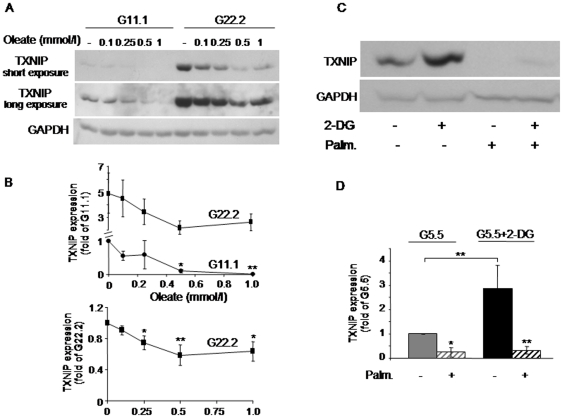
Effects of oleate and 2-deoxyglucose (2-DG) on TXNIP expression in INS-1E beta-cells. (A, B) Oleate concentration-dependent inhibition of TXNIP. INS-1E cells were incubated for 16 h with different concentrations of oleate at 11.1 (G11.1) or 22.2 (G22.2) mmol/l glucose, as indicated. TXNIP protein level was assessed by Western blot. A representative gel showing the expression of TXNIP, and quantification of TXNIP expression normalized to GAPDH are presented. Results are given as fold of TXNIP expression at 11.1 or 22.2 mmol/l glucose (G11.1 or G22.2) in the absence of oleate (B, upper and lower panels, respectively), and expressed as means ± SEM of 7 individual experiments. (C, D) Effect of 2-DG on palmitate inhibition of TXNIP. INS-1E cells were incubated at 5.5 mmol/l glucose with and without 0.5 mmol/l palmitate and 5 mmol/l 2-DG for 4 h. A representative gel showing the expression of TXNIP (C), and quantification of TXNIP expression normalized to GAPDH (D) are presented (n = 7).* p<0.05, ** p<0.01, for the difference between the indicated groups (D), and between the indicated groups and untreated cells at the same glucose concentration (B, D).

In INS-1E beta-cells palmitate increased beta-cell apoptosis at both 11.1 and 22.2 mmol/l glucose (Figure 1A). Palmitate stimulation of apoptosis was inversely related to its effect on TXNIP expression, which was decreased by palmitate in a concentration-related manner (Figure 1B, C). A time-course study showed that the inhibition of TXNIP occurred already after a 1-h incubation and progressed over time (up to 16 h) (Figure 1D, E). At both 11.1 and 22.2 mmol/l glucose, 0.5 mmol/l palmitate induced 50–70% decrease in TXNIP mRNA expression (Figure 1F), promoter activity (Figure 1G) and protein level (Figure 1B–E), indicating that palmitate inhibits TXNIP at the level of transcription. The monounsaturated long-chain fatty acid oleate, like palmitate, inhibited TXNIP expression at 11.1 and 22.2 mmol/l glucose (Figure 2A, B). There was substantial inter-experimental variation in TXNIP expression at 22.2 mmol/l compared to 11.1 mmol/l glucose. Normalization of TXNIP protein level to that at 22.2 mmol/l glucose showed that oleate inhibition of TXNIP at high glucose was significant and similar to that induced by palmitate (Figure 2B).

It was recently suggested that TXNIP expression is regulated by the glycolytic flux *via* modulation of the glucose-6 phosphate (G6P) level, which transmits the glucose signal [20]. 2-Deoxyglucose (2-DG) undergoes phosphorylation to 2-DG-6-phosphate without further metabolism, and thus can mimic the effect of G6P without the contribution of other glycolytic intermediates. Consistent with the hypothesis that early glycolytic intermediates regulate TXNIP, we found that 2-DG stimulated TXNIP like glucose (Figure 2C–D). Notably, palmitate markedly inhibited 2-DG stimulation of TXNIP, indicating that FFA inhibition of TXNIP expression is independent of glycolysis (Figure 2C–D).

**Figure 3:**
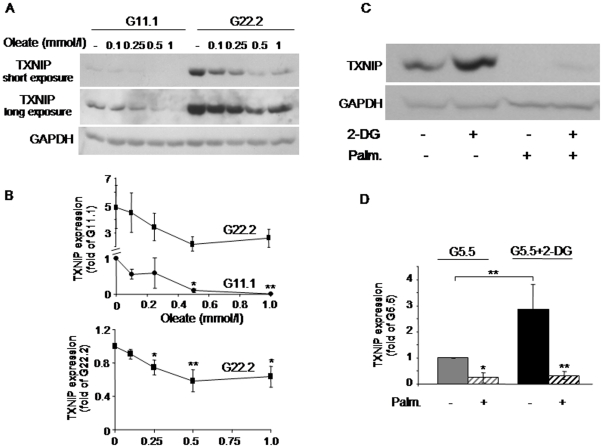
Non-metabolizable fatty acids and palmitate modulate AMPK activity and TXNIP expression in INS-1E beta-cells. (A) Effects of non-metabolizable MEDICA analogs on AMPK activity and TXNIP expression. MEDICA analogs were generated by substitution of alpha, omega-dicarboxylic acids of C16 chain length. INS-1E cells were incubated for 16 h at 11.1 (G11.1) and 22.2 (G22.2) mmol/l glucose with and without beta, beta'- or alpha, alpha'-tetramethyl-hexadecanedioic acid (M16alpha/alpha and M16beta/beta, respectively). AMPK activity was assessed by measuring phosphorylated acetyl CoA carboxylase (pACC). pACC and TXNIP protein levels were analyzed by Western blot. Quantification of TXNIP expression normalized to GAPDH (B) and ACC phosphorylation normalized to ACC (C) presented. (n = 3). (D) Effect of palmitate treatment on AMPK activity. INS-1E cells were incubated at different glucose concentrations without and with 0.5 mmol/l palmitate in KRBH buffer for 1 h. A representative gel of phospho- and total ACC and GAPDH is shown, and quantification of ACC phosphorylation presented. Results are expressed as fold of untreated cells at 3.3 mmol/l glucose (n = 10). * p<0.05, ** p<0.01, ∧ p<0.001 for the difference between MEDICA analog-treated cells and controls at the same glucose concentration (B, C), between untreated cells at different glucose concentrations and controls at 3.3 mmol/l glucose (G3.3) (D), and between palmitate-treated cells and the paired controls at the same glucose concentration (D).

The role of fatty acid beta-oxidation and/or esterification in FFA regulation of TXNIP was examined using non-metabolizable MEDICA analogs, which are neither oxidized nor esterified. These analogs were generated by substitution of alpha, omega-dicarboxylic acids of C16 chain length [19]. Beta, beta'- and alpha, alpha'-tetramethyl-hexadecanedioic acid (M16) effectively decreased TXNIP expression (Figure 3A–B), indicating that the inhibition of TXNIP by FFA is independent of esterification products or fatty acid oxidation.

cAMP is a potent inhibitor of TXNIP, in part through stimulation of protein kinase A (PKA) [21]. We further studied whether the cAMP-PKA pathway is involved in palmitate inhibition of TXNIP. INS-1E cells were treated with 3-isobutyl-1-methylxanthine (IBMX) or palmitate without and with the PKA inhibitor H89. H89 partially prevented IBMX, but not palmitate inhibition of TXNIP (Supplementary Figure S1), suggesting that FFA regulation of TXNIP is not mediated *via* cAMP-PKA.

### Association between fatty acid stimulation of AMPK and inhibition of TXNIP

**Figure 4:**
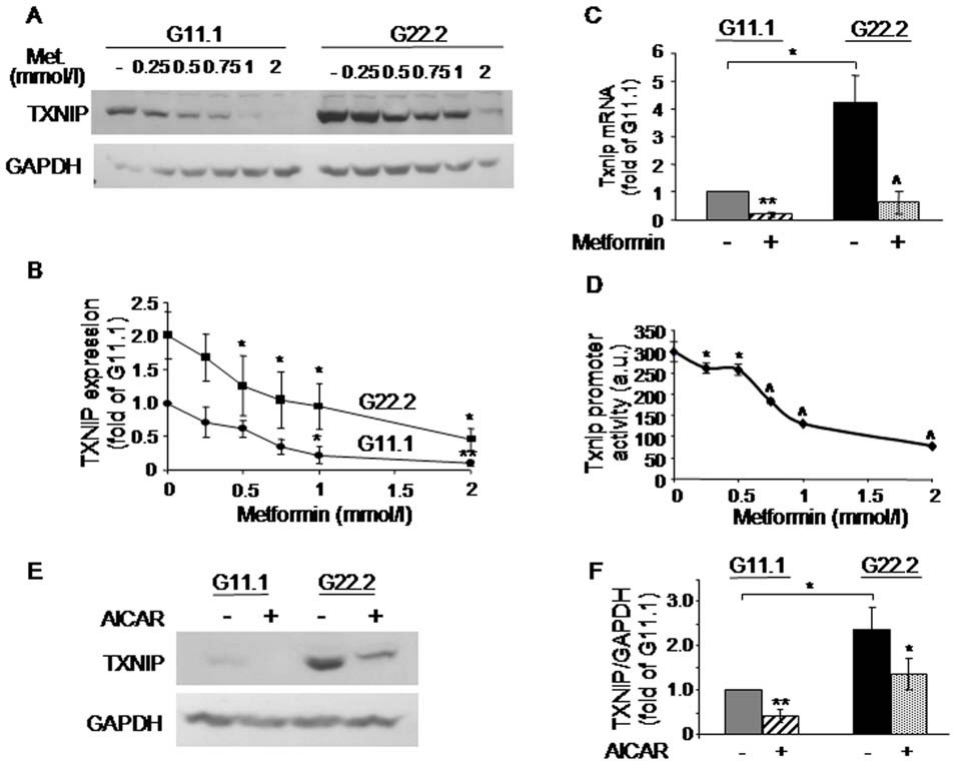
Effect of AMPK activators on TXNIP expression in INS-1E beta-cells. (A, B) Metformin concentration-dependent inhibition of TXNIP. INS-1E cells were incubated for 16 h with different concentrations of metformin at 11.1 (G11.1) or 22.2 (G22.2) mmol/l glucose. TXNIP protein level was assessed by Western blot. A representative gel for TXNIP (A) and quantification of TXNIP expression normalized to GAPDH (B) are presented. Results are expressed as fold of TXNIP expression at 11.1 mmol/l glucose in the absence of metformin (n = 3). (C, D) Metformin effect on Txnip mRNA and gene transcription. INS-1E cells were incubated in medium containing 11.1 or 22.2 mmol/l glucose with and without 1 mmol/l metformin for 16 h. (C) Txnip mRNA levels were analyzed by qPCR and corrected for the 18S rRNA, which served as an internal control (n = 5). (D) Transcriptional activity was analyzed in INS-1E cells that were stably transfected with a reporter construct encoding the human Txnip promoter region 1777 bp upstream of the ATG start codon. The cells were treated at 22.2 mmol/l glucose for 16 h with different concentrations of metformin (n = 3). (E, F) AICAR effect on TXNIP protein level. INS-1E cells were incubated in medium containing 11.1 or 22.2 mmol/l glucose with and without 1 mmol/l AICAR for 16 h. A representative blot (E) and quantification of TXNIP expression normalized to GAPDH (F), are shown. Results are expressed as means ± SEM (n = 3). * p<0.05, ** p<0.01, ∧ p<0.001 for the difference between the indicated groups and untreated cells at the same glucose concentration (B, D) or between metformin treatment (C) or AICAR treatment (F) groups and the paired control group at the same glucose concentration.

**Figure 5:**
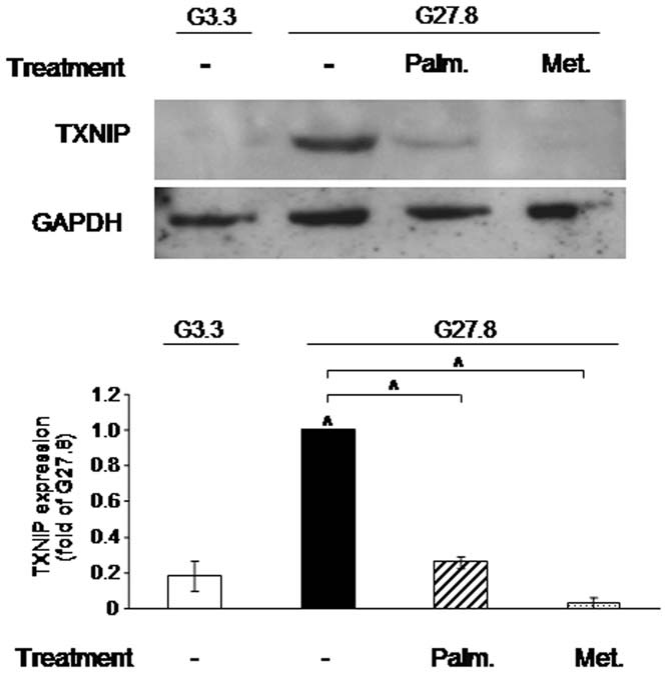
Palmitate and metformin effects on TXNIP protein levels in rat islets. Rat islets were allowed to recover overnight at RPMI medium containing at 5.5 mmol/l glucose and then incubated in medium containing 3.3 (G3.3) or 27.8 (G27.8) mmol/l glucose with and without 0.5 mmol/l palmitate (Palm.) or 1 mmol/l metformin (Met.) for 16 h. TXNIP and GAPDH protein levels were assessed by Western blot. A representative gel showing the expression of TXNIP and GAPDH, and quantification of TXNIP expression normalized to GAPDH are presented. Results are expressed as fold of TXNIP expression at 27.8 mmol/l glucose and shown as means ± SEM of 3 individual experiments, each performed on islets pooled from three animals. ∧ p<0.001 for the differences between the indicated groups or between these islets at 27.8 mmol/l glucose and control islets at 3.3 mmol/l glucose.

In agreement with previous studies [22], [23], [24], [25], high glucose decreased the AMPK activity in beta-cells, evidenced by decreased phosphorylation of the AMPK substrate ACC, whereas palmitate acutely stimulated AMPK activity (Figure 3D). Non-metabolizable MEDICA analogs are potent stimulators of AMPK (Figure 3A, C). Consistent with the hypothesis that AMPK regulates TXNIP, the degree of AMPK activation by the analogs correlated with their extent of TXNIP inhibition. To further study the role of AMPK in the regulation of TXNIP, we used two pharmacological activators of AMPK, metformin and AICAR. In INS-1E cells, metformin inhibited TXNIP transcription, gene expression and protein level in a concentration-related manner (Figure 4). Similarly, the AMPK activator AICAR also decreased the TXNIP protein level (Figure 4E, F). The inhibition of TXNIP by palmitate and metformin was confirmed in primary rat islets (Figure 5).

### Regulation of TXNIP by AMPKalpha

**Figure 6:**
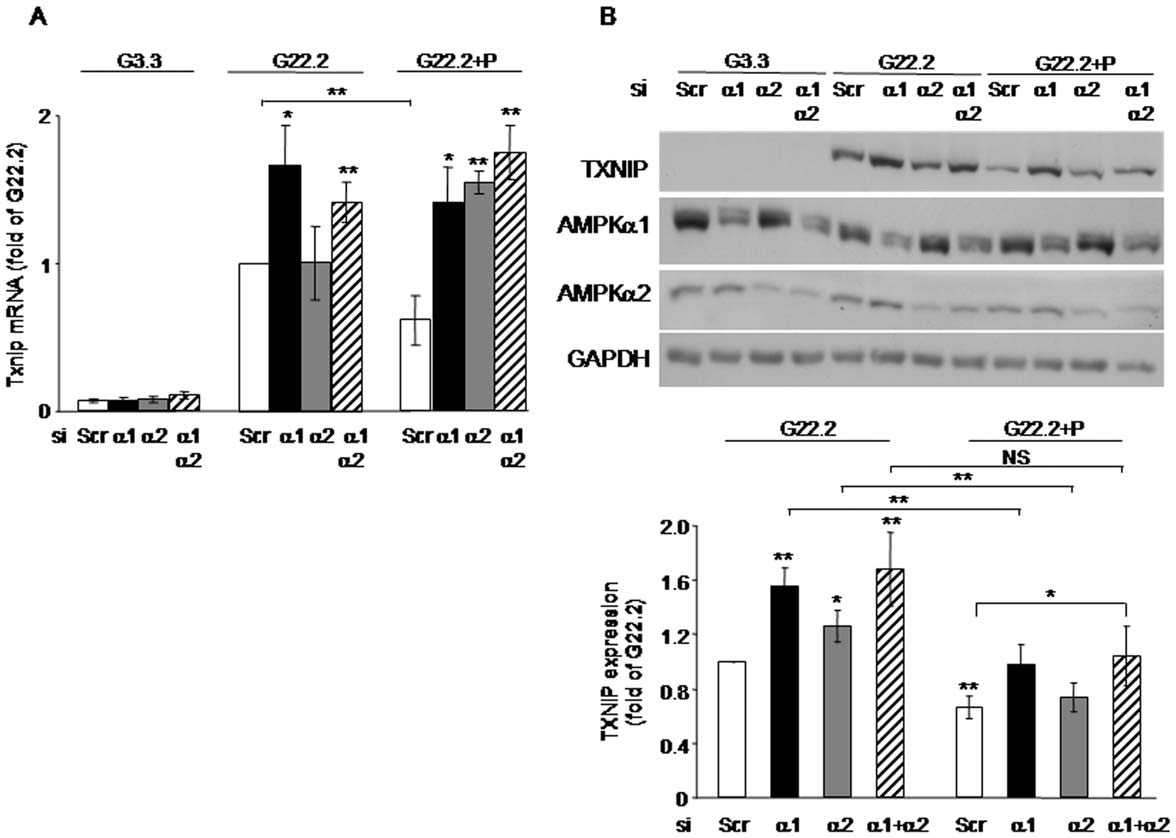
Effect of AMPKalpha isoform knockdown on TXNIP expression in INS-1E beta-cells. INS-1E cells were transfected with siRNA oligos for AMPKalpha1 (black bars), AMPKalpha2 (grey bars) or both (diagonal lines) or with control scrambled (Scr) RNA oligos (white bars) as described in the Methods. The cells were then incubated for 16 h at 3.3 (G3.3) and 22.2 mmol/l (G22.2) glucose without and with 0.5 mmol/l palmitate (P). (A) Txnip mRNA levels were analyzed by qPCR and corrected for 18S rRNA, which served as an internal control. Results are normalized to TXNIP mRNA levels at G22.2 (n = 8). (B) TXNIP, AMPKalpha1, AMPKalpha2 and GAPDH protein levels were assessed by Western blot. A representative gel and quantification of TXNIP expression normalized to GAPDH are presented. Results are expressed as fold of TXNIP expression in control cells at 22.2 mmol/l glucose and shown as means ± SEM of 11 individual experiments. * p<0.05, ** p<0.01 for the difference between the indicated groups or between similarly treated AMPKalpha knockdown cells and their controls; NS – non significant.

The catalytic activity of AMPK resides in the alpha subunit [15], which occurs as two isoforms, alpha1 and alpha2. We studied the respective role of these isoforms in nutrient regulation of TXNIP. First, we analyzed the effects of different glucose concentrations and palmitate on the expression of AMPKalpha1 and alpha2 in beta-cells. We found that total AMPK levels and the relative expression of AMPKalpha1 and alpha2 were not affected by the glucose concentration or palmitate treatment (Supplementary Figure S2). Next, we performed knockdown of the AMPKalpha isoforms and studied TXNIP expression. Knockdown of AMPKalpha1 and alpha2 increased Txnip mRNA and protein level in INS-1E cells incubated at 22.2 mmol/l glucose without and with palmitate (Figure 6). Knockdown of AMPKalpha prevented palmitate inhibition of Txnip mRNA with a smaller effect on protein level. Notably, palmitate did not decrease significantly TXNIP protein level in INS-1E cells in which AMPKalpha1 and alpha2 were silenced together. Collectively, these findings suggest that nutrients (glucose and fatty acids) affect TXNIP in part *via* modulation of AMPK activity; however, additional mechanisms are probably involved in the regulation of TXNIP protein by FFA. At 3.3 mmol/l glucose, AMPKalpha knockdown did not affect TXNIP mRNA and protein levels (Figure 6), indicating that AMPK inactivation is not sufficient for induction of TXNIP at low glucose. Furthermore, while 2-DG inhibits glycolysis, resulting in increased AMPK activity (Supplementary Figure S3), it still increased TXNIP (Figure 2C–D). Collectively, these findings suggest that the interplay between glycolytic substrate availability and AMPK activity determines the level of TXNIP expression.

### AMPK regulation of glucose-stimulated ChREBP activity

**Figure 7:**
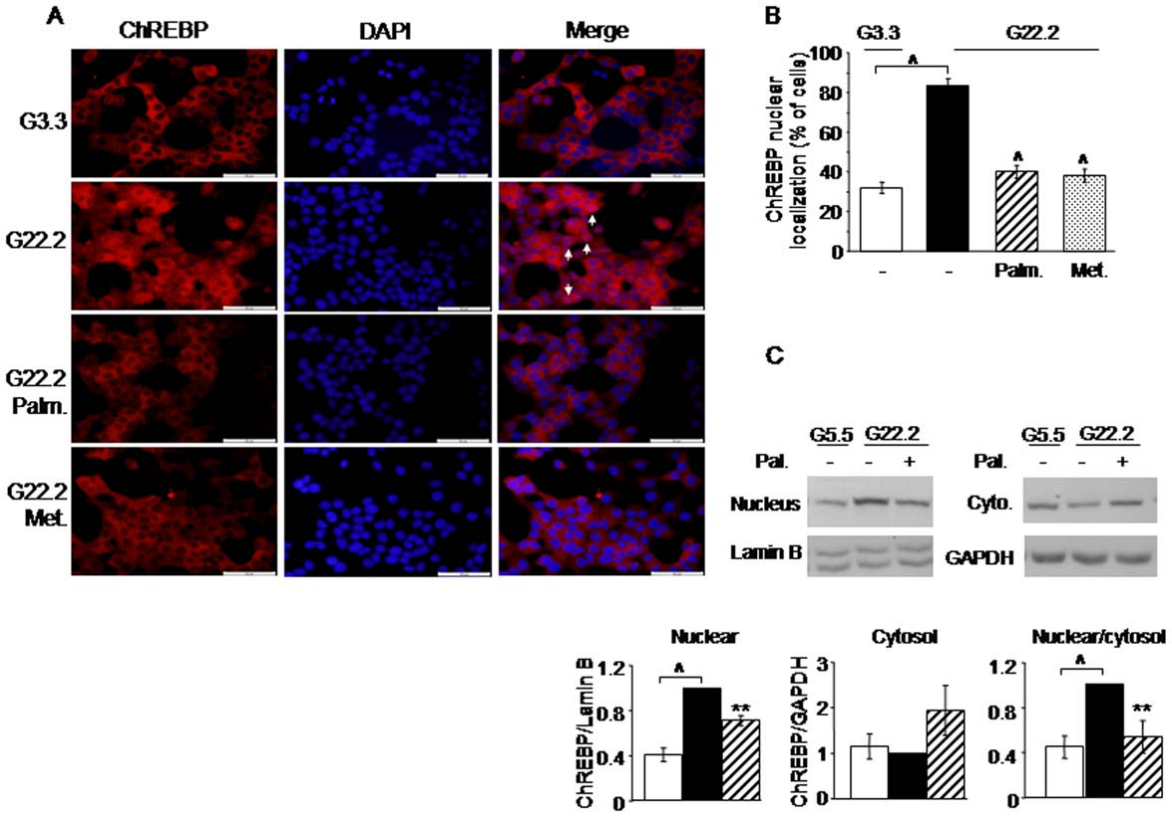
Modulation of ChREBP nuclear localization by glucose, palmitate and metformin. (A) INS-1E cells were plated on poly-D-lysine coated glass coverslips, and treated for 4 h with 3.3 (G3.3) or 22.2 (G22.2) mmol/l glucose, without or with 0.5 mmol/l palmitate or 1 mmol/l metformin. ChREBP localization was analyzed by immunofluorescence using goat antibody against human ChREBP; the secondary antibody was conjugated with rhodamine (red). Dapi was used for nuclear counterstaining (blue). Arrows denote cells with nuclear localization of ChREBP; scale bar 50 micrometer. (B) The percentage of cells showing nuclear localization of ChREBP under the different experimental conditions is shown. A minimum of 550 cells were counted. (C) ChREBP protein level was analyzed by Western blot on nuclear and cytosolic extracts of INS-1E cells treated at 5.5 (G5.5, white columns) and 22.2 (G22.2) mmol/l glucose in the absence (black columns) and with 0.5 mmol/l palmitate (Pal., diagonal columns) for 1 h. A representative blot is shown; the quantification of the nuclear and cytosolic ChREBP normalized to Lamin B and GAPDH, respectively, and the nuclear/cytosol ratios of ChREBP are also shown. Results are expressed as means ± SEM of 6 independent experiments. ** p<0.01, ∧ p<0.001 for the difference between the indicated groups and untreated cells at G22.2.

**Figure 8:**
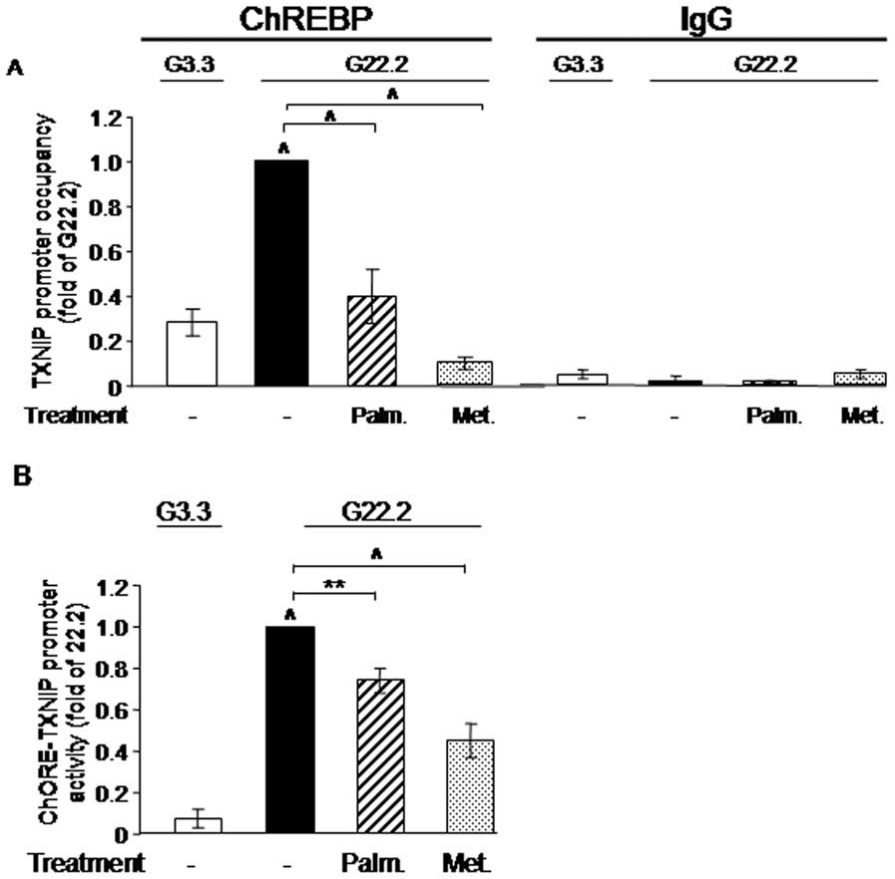
Palmitate and metformin effects on ChREBP recruitment to the Txnip promoter, and on glucose stimulation of the carbohydrate response element (ChoRE). (A) INS-1E cells were treated at 3.3 (G3.3) and 22.2 (G22.2) mmol/l glucose in the absence and with 0.5 mmol/l palmitate or 1 mmol/l metformin for 6 h. ChREBP recruitment to the Txnip promoter was studied by ChIP assay using anti-ChREBP and anti-IgG (negative control) antibodies. The ChoRE region of rat Txnip promoter was amplified by quantitative real-time RT-PCR and the percentage of occupied promoter calculated. Results are means ± SEM of 4 independent experiments. (B) Transcriptional activity was analyzed following transient transfection with a reporter construct encoding the conserved ChoRE binding site in the TXNIP promoter region 400 bp upstream of the ATG start codon. The transfected cells were then treated at 3.3 and 22.2 mmol/l glucose without and with 0.5 mmol/l palmitate or 1 mmol/l metformin for 16 h. Results are means ± SEM of 5 independent experiments. * p<0.05, ** p<0.01, ∧ p<0.001 for the difference between the indicated groups or between G22.2 and G3.3.

ChREBP plays a central role in the transcriptional regulation of TXNIP in beta-cells [11]. We studied the effects of palmitate and metformin on ChREBP nuclear localization by immunostaining and Western blot analysis (Figure 7), and on chromatin binding to the Txnip promoter by ChIP assay (Figure 8A). In INS-1E cells incubated at 3.3 mmol/l glucose, ChREBP was mostly localized in the cytoplasm. At 22.2 mmol/l glucose, the nuclear localization of ChREBP was rapidly increased (within 30 min of glucose stimulation; not shown), and remained high for at least 4 h (Figure 7A). ChREBP was found in the nuclei of ∼80% of the cells, although cytosolic expression was still present. The level of nuclear import of ChREBP varied between cells. Notably, treatment with palmitate or metformin resulted in nuclear exclusion of ChREBP, mimicking the effect of low glucose.

Chromatin immunoprecipitation (ChIP) assay (Figure 8A) showed that high glucose (22.2 mmol/l) increased ChREBP recruitment to the Txnip promoter by 4.5-fold, and that palmitate and metformin decreased ChREBP binding by 65% and 90%, respectively. Glucose-induced Txnip transcription depends on a 400 bp proximal region of the Txnip promoter, which contains a conserved ChoRE consisting of 2 non-palindromic E-boxes [26]. Transfection experiments using a luciferase reporter construct driven by the ChoRE showed, as expected, that high (22.2 mmol/l) glucose increased ChoRE-Txnip promoter activity by ∼5-fold relative to treatment with low (5.5 mmol/l) glucose; palmitate and metformin reduced the glucose stimulation of the ChoRE-Txnip promoter (Figure 8B).

Taken together, our findings show that fatty acids activate AMPK in beta-cells exposed to high glucose. Furthermore, fatty acids attenuate the glucose stimulation of ChREBP nuclear entry and its recruitment to the Txnip promoter, thereby inhibiting Txnip transcription. AMPK mediates the inhibition of TXNIP by fatty acids and is involved in TXNIP regulation by glucose.

## Discussion

In beta-cells, glucose and FFA have opposing effects on TXNIP expression. Glucose is the most potent physiological inducer of TXNIP [10], [11], [26], whereas FFA (palmitate, oleate and MEDICA analogs) function as TXNIP repressors [6]. We found that FFA inhibited TXNIP in a concentration-dependent manner, the effect being rapid in onset, progressive over time and qualitatively similar using both saturated (palmitate) and unsaturated (oleate) fatty acids. Consistent with a previous study [20], we found that increasing the level of hexose-6-phosphate by administration of 2-DG increased TXNIP. Importantly, also in the presence of 2-DG, palmitate inhibited TXNIP, indicating that the inhibitory effect of fatty acids is not due to diminution of glycolytic intermediates which regulate TXNIP, since 2DG-6P can not be metabolized further in the beta-cell. Moreover, non-metabolizable fatty acids also decreased TXNIP; thus fatty acid metabolism is not a prerequisite for the inhibition of TXNIP by FFA. The finding that palmitate inhibition of TXNIP was rapid and independent of fatty acid metabolism may suggest that this effect is mediated *via* G-protein coupled receptors (GPCR). Long-chain fatty acids and their derivatives may bind to GPCR, which mediate their effects on important biological processes in the beta-cell including insulin secretion [27]. Future studies will clarify whether GPCR mediate FFA inhibition of TXNIP and which receptor is involved in this process.

Our findings strongly suggest that nutrient regulation of TXNIP is mediated *via* AMPKalpha. At high glucose, AMPK activity is decreased; however this inhibition is incomplete. The residual activity of AMPK in hyperglycemia restrains TXNIP expression, as its knockdown augmented glucose stimulation of TXNIP. The observation that the AMPK activators metformin and AICAR markedly decreased TXNIP expression at high glucose further supports the role of AMPK in the regulation of TXNIP under conditions of hyperglycemia. Contrasting the effect of glucose, FFA stimulated AMPK and decreased TXNIP expression. Importantly, AMPKalpha silencing prevented the inhibition of TXNIP by fatty acid, thus stressing the central mediator role of AMPKalpha in this regulatory loop. AMPK is not the exclusive factor regulating TXNIP in response to changes in nutrient availability. This claim is based on the following observations: 1. AMPKalpha knockdown failed to induce TXNIP at low glucose; 2. 2-DG stimulates TXNIP, while inhibiting glycolysis with simultaneous activation of AMPK. Thus, the regulation of TXNIP by nutrients is complex and influenced by their impact on glycolytic substrate availability and on signaling pathways, including AMPK.

Gene expression analysis and transfection experiments clearly showed that FFA inhibited TXNIP at the level of transcription. The transcription factor ChREBP has been shown recently to play a central role in glucose stimulation of TXNIP [11]. Consistent with this, we found that glucose rapidly increased the nuclear localization of ChREBP and its recruitment to the Txnip promoter. Uyeda and Repa [28] showed that ChREBP activity is regulated through phosphorylation-dependent mechanisms, which affect its subcellular localization and DNA binding. According to this model, phosphorylation of ChREBP residues Ser-196, Ser-626 and Thr-666 at low glucose restricts it to the cytoplasm, thereby inhibiting its activity [29]. Glucose activates the phosphatase PP2A leading to dephosphorylation of ChREBP at these sites, thus promoting its nuclear localization and DNA binding [30]. This model, however, has been challenged by others [31], [32], [33], [34]. We found that palmitate reduced ChREBP nuclear localization in response to glucose. The precise mechanism is currently unknown; yet, this is expected to inhibit ChREBP activity. Indeed, the decrease of ChREBP nuclear localization was accompanied by inhibition of ChREBP-stimulated Txnip transcription. Moreover, metformin decreased both ChREBP nuclear localization and its chromatin binding to the Txnip promoter. Yet, AMPK may affect ChREBP chromatin binding directly, independent of its nuclear localization. Consistent with this hypothesis, high-fat feeding was previously shown to increase liver AMPK activity, leading to ChREBP phosphorylation and consequently to inhibition of its DNA binding, without affecting its nuclear localization [35]. It is noteworthy that cAMP also was shown to repress ChREBP activity through PKA-mediated phosphorylation adjacent to the nuclear localization signal of the molecule [29], [36]. This promotes interactions with 14-3-3 proteins and thus sequesters ChREBP in the cytosol [37]. Others and we have previously shown that GLP-1 and the phosphodiesterase inhibitor IBMX, which raise cAMP levels, inhibited TXNIP expression in beta-cells [12], [38]. Taken together, these findings suggest that AMPK- and cAMP/PKA-mediated ChREBP phosphorylations provide an important mechanism for the regulation of TXNIP expression. Microinjection of antibodies against AMPKalpha2 increased proinsulin and L-type pyruvate kinase (L-PK) gene transcription in beta-cells, mimicking the glucose effects [39]. The finding that inhibition of AMPKalpha enhanced the expression of *bona fide* ChREBP targets, such as L-PK [28] and TXNIP (this study), suggests that AMPKalpha-ChREBP orchestrates a genetic network implicated in nutrient metabolism and oxidative stress.

TXNIP inhibits glucose uptake in different tissues [40], [41], thus suppression of TXNIP by FFA may promote glucose uptake, thereby ensuring adequate glycolytic intermediates for effective fatty acid metabolism. However, in contrast to muscle and fat, glucose uptake in the beta-cell is mediated *via* glucose transporter 2 (GLUT2), which has a low affinity for glucose, and is thus not a regulatory step in beta-cell glucose metabolism [42]. Therefore this hypothesis seems to be less likely. Palmitate is toxic to beta-cells; the finding that FFA inhibit rather than increase TXNIP supports the suggestion that lipotoxicity is not mediated through oxidative stress induced by inhibition of the thioredoxin system. Palmitate stimulation of beta-cell apoptosis paralleled its inhibition of TXNIP expression (Figure 1). Therefore, palmitate inhibition of TXNIP may be considered an adaptive defense mechanism in the face of glucolipotoxicity-induced cellular stress, aimed to reduce its impact on beta-cell function and survival. It might be expected that activation of AMPK and consequently inhibition of TXNIP would improve beta-cell survival. However, the role of AMPK in beta-cell survival is highly controversial. Several studies showed that activation of AMPK could lead to beta-cell apoptosis [43], [44], and that AMPK inhibition may protect beta-cells from immune-mediated death [45]. On the other hand, others showed that nitric oxide (NO) activated AMPK, which then suppressed apoptotic signals, allowing the beta-cells to recover from nitrosative stress [46]. Consistent with this, we found that NO inhibited TXNIP expression, whereas NOS inhibition increased it [12]. The conflicting results regarding the role of AMPK in beta-cell survival could result from the complex regulation of multiple metabolic and stress pathways by AMPK including, but not limited to, TXNIP expression. Thus, the impact of AMPK activation on beta-cell survival seems to vary depending on the metabolic context and the cellular system used; this may explain the finding that AMPK activation is not consistently beneficial for beta-cell survival despite TXNIP inhibition.

In summary, we found that fatty acids are negative regulators of TXNIP, the effect being mediated *via* AMPKalpha, leading to ChREBP inactivation with reduced Txnip transcription. Under conditions of hyperglycemia, residual AMPKalpha activity restricts the TXNIP stimulation by glucose. Thus, AMPK plays a crucial role in the regulation of TXNIP by nutrients. These findings may have important implications for the development of novel therapeutic strategies for diabetes based on TXNIP inhibition.

## Supporting Information

Figure S1 **cAMP-PKA regulation of TXNIP in INS-1E beta-cells.** INS-1E cells were incubated for 4 h at 22.2 mmol/l glucose with 0.1 mmol/l 3-isobutyl-1-methylxanthine (IBMX) or 0.5 mmol/l palmitate, without or with the protein kinase A (PKA) inhibitor H89 (10 micromol/l). Cells treated with H89 were pre-incubated with the inhibitor for 30 minutes. TXNIP expression was analyzed by Western blot. A representative experiment and quantification of TXNIP expression normalized to GAPDH are shown. Results are expressed as means ± SEM (n = 4). ∧ p<0.001 for the difference between the IBMX treatment group and control (untreated cells at G22.2), and * p<0.05 for the difference between the IBMX treatment groups without and with H89; NS- non significant.
(TIF)

Figure S2 **Glucose and palmitate effects on total AMPK and AMPKalpha isoform protein level (A–B) and gene expression (C–D).** INS-1E cells were incubated at 11.1 or 22.2 mmol/l glucose with and without 0.5 mmol/l palmitate for 3, 6 and 16 h. AMPKalpha1 and AMPKalpha2 protein levels were analyzed by Western blot (A, B). A representative gel (A) and quantification of AMPKalpha isoform expression at 16 h normalized to control protein levels at 3.3 mmol/l glucose (B) are shown. AMPKalpha isoform expression was normalized to GAPDH. AMPK isoform mRNA levels were analyzed by qPCR and normalized to mRNA levels at 3.3 mmol/l glucose (G3.3) (C–D). Results are expressed as means ± SEM (n = 3).
(TIF)

Figure S3 **Effects of 2-deoxyglucose (2-DG) on AMPK activity.** INS-1E cells were incubated at 5.5 mmol/l glucose without and with 5 mmol/l 2-DG for 4 h. AMPK activity was assessed by measuring phosphorylated acetyl CoA carboxylase (pACC). A representative gel of phospho- and total ACC and quantification of 4 independent experiments is shown. Results are expressed as fold of untreated cells. * p<0.05 for the difference between the 2-DG treatment group and untreated controls.
(TIF)
