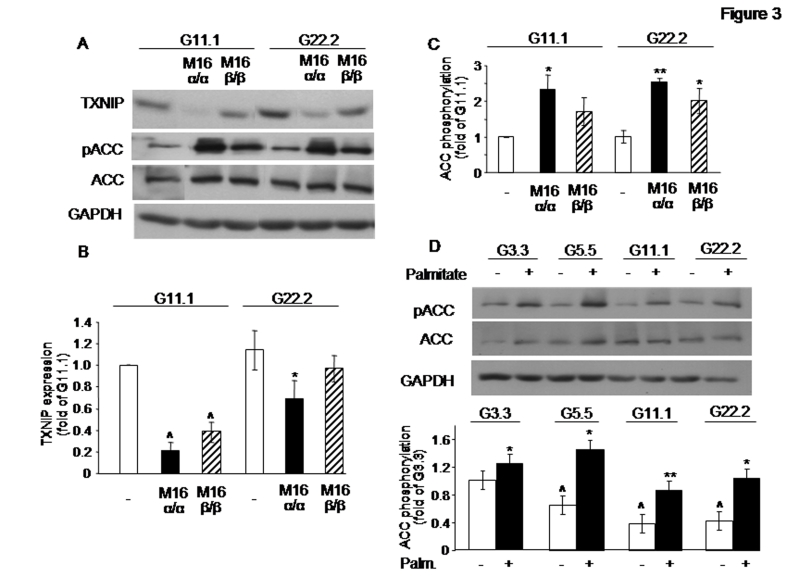# Correction: AMP-Activated Protein Kinase (AMPK) Mediates Nutrient Regulation of Thioredoxin-Interacting Protein (TXNIP) in Pancreatic Beta-Cells

**DOI:** 10.1371/annotation/23f84cc3-627d-4012-ae6f-f3291b2567d0

**Published:** 2012-01-28

**Authors:** Maayan Shaked, Mali Ketzinel-Gilad, Erol Cerasi, Nurit Kaiser, Gil Leibowitz

There was an error in Figure 3. Figure 3 was identical to Figure 2. The correct Figure 3 can be viewed here: 

**Figure:**