# Supplementary figures and images for: AMP-Activated Protein Kinase (AMPK) Mediates Nutrient Regulation of Thioredoxin-Interacting Protein (TXNIP) in Pancreatic Beta-Cells
Source: PLoS One. 2011 Dec 14;6(12):e28804. doi: 10.1371/journal.pone.0028804 (PMC3237554; doi:10.1371/journal.pone.0028804)

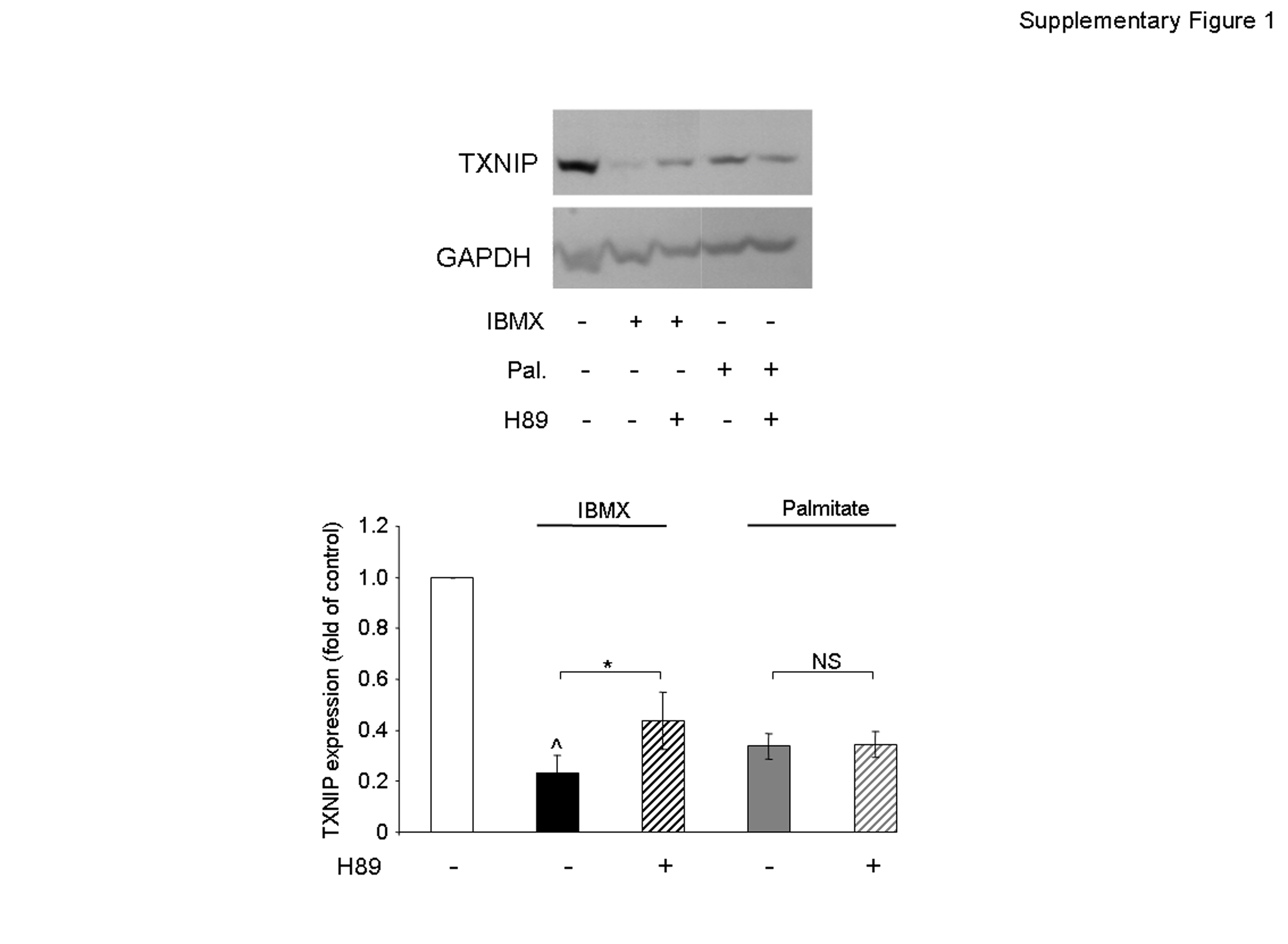

Supplement: Figure S1 — cAMP-PKA regulation of TXNIP in INS-1E beta-cells. INS-1E cells were incubated for 4 h at 22.2 mmol/l glucose with 0.1 mmol/l 3-isobutyl-1-methylxanthine (IBMX) or 0.5 mmol/l palmitate, without or with the protein kinase A (PKA) inhibitor H89 (10 micromol/l). Cells treated with H89 were pre-incubated with the inhibitor for 30 minutes. TXNIP expression was analyzed by Western blot. A representative experiment and quantification of TXNIP expression normalized to GAPDH are shown. Results are expressed as means ± SEM (n = 4). ∧ p<0.001 for the difference between the IBMX treatment group and control (untreated cells at G22.2), and * p<0.05 for the difference between the IBMX treatment groups without and with H89; NS- non significant. (TIF) [file pone.0028804.s001.tif]

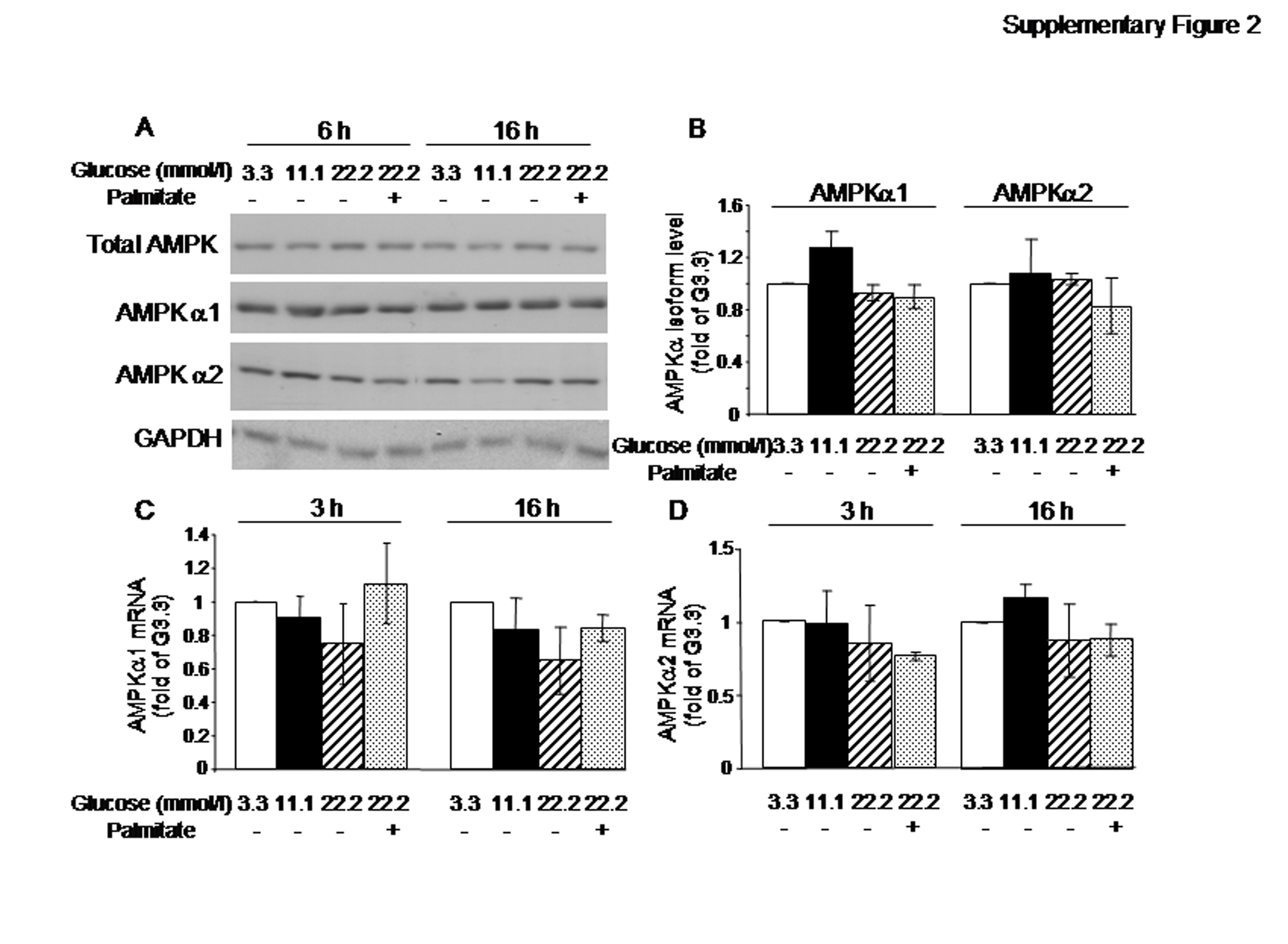

Supplement: Figure S2 — Glucose and palmitate effects on total AMPK and AMPKalpha isoform protein level (A–B) and gene expression (C–D). INS-1E cells were incubated at 11.1 or 22.2 mmol/l glucose with and without 0.5 mmol/l palmitate for 3, 6 and 16 h. AMPKalpha1 and AMPKalpha2 protein levels were analyzed by Western blot (A, B). A representative gel (A) and quantification of AMPKalpha isoform expression at 16 h normalized to control protein levels at 3.3 mmol/l glucose (B) are shown. AMPKalpha isoform expression was normalized to GAPDH. AMPK isoform mRNA levels were analyzed by qPCR and normalized to mRNA levels at 3.3 mmol/l glucose (G3.3) (C–D). Results are expressed as means ± SEM (n = 3). (TIF) [file pone.0028804.s002.tif]

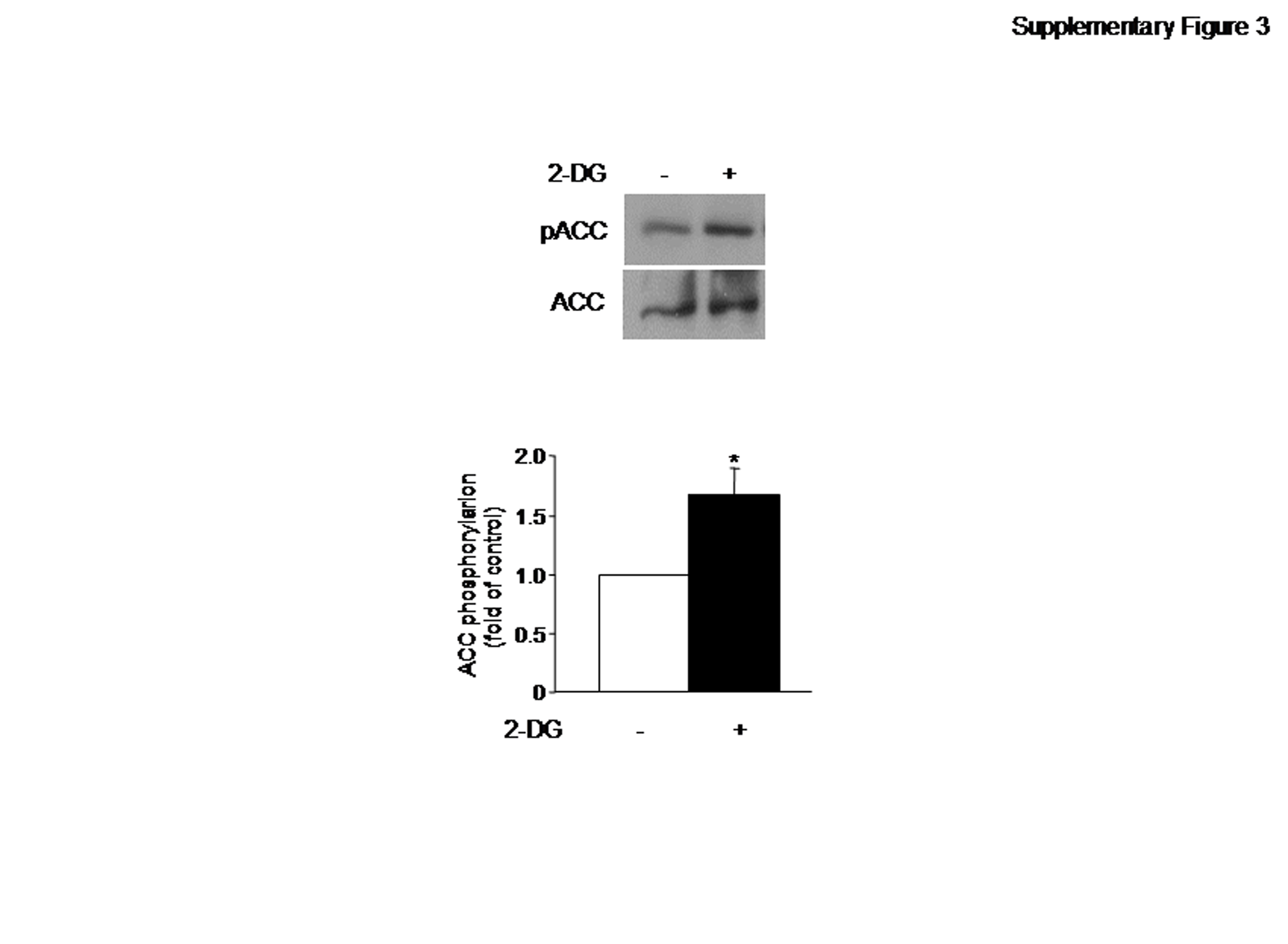

Supplement: Figure S3 — Effects of 2-deoxyglucose (2-DG) on AMPK activity. INS-1E cells were incubated at 5.5 mmol/l glucose without and with 5 mmol/l 2-DG for 4 h. AMPK activity was assessed by measuring phosphorylated acetyl CoA carboxylase (pACC). A representative gel of phospho- and total ACC and quantification of 4 independent experiments is shown. Results are expressed as fold of untreated cells. * p<0.05 for the difference between the 2-DG treatment group and untreated controls. (TIF) [file pone.0028804.s003.tif]
